# Whole blood genome-wide transcriptome profiling and metagenomics next-generation sequencing in young infants with suspected sepsis in a low-and middle-income country: A study protocol

**DOI:** 10.12688/gatesopenres.13172.2

**Published:** 2020-11-26

**Authors:** Constantin R. Popescu, Bentry Tembo, Rhoda Chifisi, Miranda M.M. Cavanagh, Amy Huei-Yi Lee, Blessings Chiluzi, Emily J. Ciccone, Gerald Tegha, Esther Alonso-Prieto, Jennifer Claydon, Dustin Dunsmuir, Mike Irvine, Guy Dumont, J. Mark Ansermino, Matthew O. Wiens, Jonathan J. Juliano, Niranjan Kissoon, Tisungane Mvalo, Norman Lufesi, Msandeni Chiume-Kayuni, Pascal M. Lavoie

**Affiliations:** 1 BC Children’s Hospital Research Institute, Vancouver, BC, Canada; 2 Department of Pediatrics, Université Laval, Québec, QC, Canada; 3 Kamuzu Central Hospital, Lilongwe, Malawi; 4 BC Children’s & Women’s Health Centre, Vancouver, BC, Canada; 5 Department of Molecular Biology and Biochemistry, Simon Fraser University, Burnaby, BC, Canada; 6 Department of Medicine, University of North Carolina, Chapel Hill, NC, USA; 7 University of North Carolina Project Malawi, Lilongwe, Malawi; 8 Department of Pediatrics, University of British Columbia, Vancouver, BC, Canada; 9 Department of Anesthesiology, Pharmacology & Therapeutics, University of British Columbia, Vancouver, BC, Canada; 10 Department of Electrical and Computer Engineering, University of British Columbia, Vancouver, BC, Canada; 11 Walimu, Kampala, Uganda; 12 Mbarara University of Science and Technology, Mbarara, Uganda; 13 Division of Infectious Diseases, School of Medicine, University of North Carolina, Chapel Hill, NC, USA; 14 Department of Epidemiology, Gillings School of Global Public Health, University of North Carolina, Chapel Hill, NC, USA; 15 Curriculum in Genetics and Molecular Biology, School of Medicine, University of North Carolina, Chapel Hill, NC, USA; 16 Department of Pediatrics, University of North Carolina, Chapel Hill, NC, USA; 17 Clinical Services Directorate, Ministry of Health, Lilongwe, Malawi

**Keywords:** Newborns, young infants, sepsis, prospective study, low- and middle-income country, epidemiology, diagnosis, RNA sequencing, metagenomics, blood culture.

## Abstract

Conducting collaborative and comprehensive epidemiological research on neonatal sepsis in low- and middle-income countries (LMICs) is challenging due to a lack of diagnostic tests. This prospective study protocol aims to obtain epidemiological data on bacterial sepsis in newborns and young infants at Kamuzu Central Hospital in Lilongwe, Malawi. The main goal is to determine if the use of whole blood transcriptome host immune response signatures can help in the identification of infants who have sepsis of bacterial causes. The protocol includes a detailed clinical assessment with vital sign measurements, strict aseptic blood culture protocol with state-of-the-art microbial analyses and RNA-sequencing and metagenomics evaluations of host responses and pathogens, respectively. We also discuss the directions of a brief analysis plan for RNA sequencing data. This study will provide robust epidemiological data for sepsis in neonates and young infants in a setting where sepsis confers an inordinate burden of disease.

## Introduction

Sepsis is defined as life-threatening organ dysfunction caused by a dysregulated host response to infection ^1^. Each year, over 11 million people die of sepsis worldwide, with young infants accounting for nearly a quarter of these deaths ^2– 4^. By far, the greatest burden of infants’ sepsis mortality occurs in low- and middle-income countries (LMIC). Concerted global efforts have resulted in a 50% reduction in sepsis-related mortality in children below 5 years of age, over three decades. However, health gains in young infants lag behind older children ^5^. Furthermore, precise epidemiological data on the incidence and risk factors for neonatal sepsis are still lacking especially in LMIC where the vast majority of cases occur ^2, 3, 6, 7^.

Effective public health measures and treatment guidelines rely on robust evidence and case identification using clear diagnostic criteria, both of which are lacking in young infants ^8^. This makes the management of sepsis in this age group challenging. In young infants, sepsis can be due to a variety of pathogens including viruses (e.g. parechovirus infection, herpes infection), fungi (e.g. *Candida*) and bacteria (including usual pathogens, but potentially bacteria that are generally associated with normal commensals on the skin and contamination of cultures). Early clinical signs are inconspicuously non-specific and often overlap with other health conditions, making sepsis difficult to recognize based on clinical criteria alone. This is compounded by the need for prompt antibiotic treatment to ensure survival from bacterial sepsis in infants. Currently, blood cultures are the gold standard for diagnosis of bacterial sepsis. However, the specificity and sensitivity of blood cultures are low in young infants. Also, blood culture contamination can be frequent in low resource settings due to a lack of resources and disinfection policies ^9^. As such, positive blood culture results do not necessarily indicate a true infection because of the high possibility of sample contamination during blood collection when aseptic procedures are not strictly followed. Furthermore, differentiating a blood culture contamination from a true infection can be problematic particularly due to the common occurrence of bacteremia involving commensal skin pathogens in newborns ^10^. In addition to limiting the epidemiological understanding of the problem, these challenges greatly complicate the management of infants with sepsis in LMICs ^11^ and seriously limit the development of effective prevention and treatment guidelines for antibiotic use.

Ribonucleic acid (RNA) profiling using deep sequencing in whole blood detects the host immune response produced during an infection. This approach provides useful information about the etiology and, potentially, the severity of sepsis in humans. Studies in the United Kingdom ^12, 13^, Spain ^14^ and the United States of America ^15, 16^ have shown that host transcriptome signatures can discriminate between bacterial and viral causes of sepsis, as well as other syndromes in infants under 3 months of age. To date, very few studies have used RNA-sequencing (RNA-Seq) for case identification of bacterial sepsis in neonates, and to understand the etiology of sepsis in LMIC which globally represent over 90% of the disease burden. Therefore, we hypothesize that RNA-Seq of host immune responses in whole blood will inform the “true” prevalence and epidemiology of bacterial sepsis in infants in a LMIC setting. In addition, we hypothesize that metagenomic next-generation sequencing (mNGS) technologies may be useful in this study population for detection of etiologic pathogens and genes conferring antimicrobial resistance (AMR) further augmenting our epidemiological knowledge ^17– 20^.

To help fill these knowledge gaps, we developed a prospective study protocol that aims to:

1) Determine the prevalence of bacterial sepsis in infants under three months evaluated for suspected sepsis at a regional hospital in Lilongwe, Malawi, Africa.

2) Establish whether blood molecular RNA signatures in this setting can more accurately identify young infants with bacterial causes among those in whom sepsis is suspected.

3) Provide proof-of-concept that mNGS can detect pathogens and AMR genes in infants with sepsis.

## Study protocol

### Study design

This will be a prospective, longitudinal cohort study.

### Setting

Kamuzu Central Hospital (KCH), based in Lilongwe, is the largest referral hospital for the central region of Malawi (population ~18 millions), delivering adult and pediatric clinical care for ~5 million inhabitants. At KCH, infants under 2 weeks of age are admitted to a dedicated Neonatal Unit from the maternity ward, home, or another hospital facility. Infants between 2 weeks and 3 months are admitted from home or another hospital to the Special Care Nursery in the main pediatric ward or a High-Dependency Unit if they require oxygen therapy.

### Participants

Infants less than 3 months with suspected sepsis from all gender and ethnic groups who present to the Neonatal Unit, pediatric Special Care Nursery or High-Dependency Unit at KCH are eligible for inclusion if they have received antibiotics for less than 4 hours prior to enrollment. The 4-hour cut-off was extensively discussed within our study group. First, based on experience we expect that only a small proportion of infants will have received antibiotic treatment for less than 4 hours prior to initial presentation in this setting. We also considered data suggesting that blood cultures positivity rapidly declines after antibiotic administration ^21^. Finally, we discussed with our Malawian colleagues that it could be ethically challenging not to offer the study to infants who have received antibiotics for less than 4 hours considering that these infants may benefit from the information provided by blood cultures that were only available as part of this study protocol. Beyond 4 hours, we estimated that the benefit of blood cultures would be sufficiently low to ethically and scientifically justify excluding those infants.

A separate group of infants less than 3 months of age without concerns for sepsis and who require blood sampling for any clinical indications (justifying an extra research sample) will be included as *controls* for the transcriptome or mNGS analyses. Informed consent will be obtained from these infants using the same form. Because antibiotic use will be recorded throughout the entire hospitalization, we will be able to exclude infants who appear well initially but become ill during admission from the control group. Therefore, the risk of misclassification is likely negligeable. Written informed consent (see Consent Form in *Extended data* ^22^) will be obtained from parents/legal guardians in English or Chichewa (local dialect).

### Study procedures

Active recruitment will begin in June 2018, after a 2-week period of study training with the Malawi team. Infants will be screened daily for eligibility at the time of initial presentation for suspected sepsis. Eligibility will be determined by a trained study nurse or clinical officer on-site, who will also obtain consent in English or Chichewa, collect the history of the presenting illness and perform a physical examination (with vital signs), following a pre-specified Data Collection Form (see *Extended data* ^22^). Consent will take place in a location as private as possible, near the bedside or in a separate room. Acknowledging the typical congestion in the hospital environment, complete privacy is not always possible. Research staff will be trained in International Conference on Harmonization Good Clinical Practices and will follow the highest possible standards of privacy and confidentiality.

A log of all infants approached for consent will be collected. Additionally, unit census data from all admissions will be reviewed at the end of the study, to determine the overall number of eligible infants. For participants, a separate paper log of participant ID and name will be maintained.

Once consent has been granted, additional verbal permission will be obtained from parents to record a short video (~30 seconds) of the infant, using a High Definition iPad camera. Vital signs (heart rate, respiratory rate, oxygen saturation, temperature and blood pressure) will be recorded by a study nurse or clinical officer on admission, and daily thereafter by a dedicated vital sign assistant. Heart rate, respiratory rate and oxygen saturation will be captured using a custom application developed by the Digital Health Innovation Lab at the BC Children’s Hospital Research Institute and Centre for International Child Health (DD, GD, JMA) that employs a saturation probe connected to an Android device ^23^. Blood pressure will be measured via automated monitors (General Electric Dinamap Pro 300V2 monitor) using a standard blood pressure protocol (see *Extended data* ^22^). Axillary body temperatures will be obtained using electronic thermometers (Welch Allyn SureTemp, model 692) by trained staff. Blood pressure monitors and electronic thermometers were provided by the study investigators at the beginning of the study.

A complete blood count with differential, blood glucose, blood culture, a urine culture by catheter and a lumbar puncture (as determined by the medical team) will be collected at the time of initial assessment, as per the standard of care for infants with suspected sepsis in Malawi ^24^. A blood sample (0.5 mL) for RNA studies will also be collected in RNA *later* ^*TM*^ (Invitrogen), at the time of blood sampling to minimize infants’ discomfort. An additional 2 mL of blood will be collected in 100 infants weighing more than 2.5 kg (for safety reasons, as the research blood sample in smaller infants was deemed to be too invasive), in addition to a rectal swab from the infant, and a vaginal swab from the mother, for mNGS analyses.

Prior to initiating the study, clinical staff will be trained on a specific blood culture sampling protocol (see *Extended data* ^22^) designed and implemented in conjunction with the clinical team at KCH to minimize blood culture contamination and provide at least 2 mL of blood for pathogen detection.

Blood will be inoculated into BACTEC PEDS Plus bottles, incubated in a BD BACTEC 9050. Cerebrospinal fluid samples will be plated to sheep blood and chocolate agar media and a thioglycollate broth tube and incubated for five days. Gram stain (Fisher Healthcare protocol Gram stain set with stabilized iodine) will be performed according to manufacturer.

Blood culture bottles flagged by the instrument as possible growth will be further analyzed by Gram stain performed on the sample. Samples will be plated to appropriate media based on organism morphology seen on the Gram stain. Identification of organisms for cultures of cerebrospinal fluid with growth will be completed using biochemical tests, bioMerieux API, and BD Crystal kits. Antimicrobial susceptibility testing will be performed by disk diffusion (BD BBL Susceptibility Disks) and/or MIC (bioMerieux E-Test) in accordance with Clinical and Laboratory Standards Institute (CLSI) M100 Performance Standards for Antimicrobial Susceptibility Testing guidelines and according to manufacturer. If no growth is detected after five days, the culture result will be finalized.

Complete blood counts with cell differential will be performed in an EDTA whole blood sample using a Beckman Coulter AcT5 Diff analyzer. Samples will be tested within 24 hours of collection. Samples that were clotted or demonstrated 3–4+ hemolysis will be rejected.

The processing of blood counts, blood culture and cerebrospinal fluids (as indicated), as well as the storage (-80°C) of the RNA-protected research whole blood samples, will be done on-site by the University of North Carolina (UNC)-Project Malawi laboratory. At the end of the study, research blood samples and all bacterial isolates will be shipped in a single batch, on dry ice to Vancouver, Canada.

The standard of treatment for sepsis at KCH ** is to use intravenous (IV) benzylpenicillin 50,000 International Units (IU) per kg of body weight twice a day for neonates younger than 7 days and 4 times a day for infants between 7 days and 3 months of age. In addition, IV gentamicin is used at 3 mg per kg of body weight once a day for low birth weight infants, or 5 mg per kg of body weight once a day in appropriately grown term infants, and 7.5 mg per kg of body weight once per day for infants between 7 days and 3 months of age. The total duration of antibiotic treatment is dependent on the clinical course. If the infant is able to tolerate feeds orally, is afebrile and otherwise clinically well, oral antibiotics are administered after 3 days of IV treatment, using either amoxicillin 125 mg every 8 hours or erythromycin 125 mg every 6 hours for an additional 5 days. In cases of atypical pneumonia, azithromycin 10 mg once per day for 3 days is used. For suspected meningitis, the dose of penicillin is increased (100,000 IU – same dosing interval). Intravenous ceftriaxone 100 mg per kg once a day is used as a second-line antibiotic treatment if there is no response to the first-line, or in cases of suspected meningitis. During hospitalization, clinical interventions, including antibiotic treatment will be provided as per the medical team and will follow the standard of care at KCH ^24^.

### Ethical considerations

The study received approval on October 3, 2017 from the National Health Sciences Research Committee at the Ministry of Health in Lilongwe, Malawi (under study #17/8/1819; title: “Improving the Early Diagnosis of Neonatal Sepsis”, amended Oct 4th, 2019 to include mNGS in a subgroup of infants), and on October 18, 2017 from the UBC Children’s & Women’s Research Ethics Board (certificate #H16-02639; Vancouver, Canada).

The study will provide a standardized blood culture and complete blood count with differential to all participants, as these tests are often not available clinically due to laboratory resource limitations. Cerebrospinal fluid and urine cultures will also be provided whenever the clinical team determines these tests are indicated as per the standards of clinical care in Malawi.

### Data collection

Data will be prospectively collected at the time of presentation and during hospitalization until final disposition, as detailed in the Data Collection Form (see *Extended data* ^22^), using standardized paper and electronic forms. Variables are designed to be largely self-explanatory with no attempt made at pre-specifying definitions. However, all history and physical exam data will be captured by clinical staff members trained to the study protocol during the 2-week run-in period, under the supervision of a single clinical officer (BT). Gestational maturity will be estimated visually using a Ballard assessment (for newborns) when the information cannot be provided from the caregiver or the chart.

De-identified data will be entered into a password-protected REDCap database using a password-protected iPad device ^25^. Electronic REDCap data (including videos) will be uploaded weekly via a dedicated wi-fi network onto a secured database hosted at the BC Children’s Hospital Research Institute (Vancouver, Canada). No information that discloses the identity of participants will be recorded on the mobile study devices during data collection. No personal information will be published. A list of study personnel and their delegated tasks will also be maintained by the study coordinator in Vancouver.

During the study, paper-based data collection forms, including consent forms, will be stored at KCH in a secured place, under the responsibility of the site PI (MC). Access to the data will be limited to co-investigators and study members directly involved in the study via secured access to the main server in Vancouver, Canada. At the end of the study, records will be reviewed and the data verified by at least two study investigators for accuracy and completeness. All study-related documents will be kept for at least 5 years according to policies from the University of British Columbia. De-identified data will be made publicly available, following approval by the Vancouver and Malawi research ethics boards.

### Partnerships

Study staff will be hired from a pool of clinical staff dedicated to the neonatal unit at KCH, through a partnership with the Pediatric and Child Health Initiative (PACHI). The metagenomic sequencing for pathogen and AMR gene detection sub-study component is conducted in partnership with UNC Project-Malawi and the UNC. MNGS will be analyzed through the Chan-Zuckerberg Initiative.

### Study size

Precise *a priori* power calculations are difficult due to the absence of transcriptome data in similar LMIC cohorts. Also, there are no published data on the incidence of bacterial neonatal sepsis at KCH. However, based on communications with local study investigators, we expect that ~20% of the infants in the study will have a positive blood culture. Therefore, we estimate that enrolling 300 infants will yield about 60 bacterial sepsis cases. This will provide 80% power to detect differences in expression for ~40 gene markers, considering previous studies ^26, 27^, using a 5% false-discovery rate method of adjustment for multiple comparisons. Based on studies conducted at the Queen Elizabeth Central Hospital in Blantyre (the other regional referral center in Malawi), we also expect relatively high resistance to first line antimicrobials for Gram-negative bacteria ^28^. An additional 100 infants will be enrolled for the mNGS objective. As this study component is exploratory, no formal sample size calculation was performed for the mNGS sub-study.

### Data analysis

Data will be coded to facilitate analysis. The cohort will initially be analyzed descriptively, listing baseline demographic and clinical variables with mean ± standard deviation, median with interquartile range, and proportions (with 95% confidence intervals) depending on the data distribution. Bacterial species and antimicrobial resistance patterns for positive blood cultures will also be reported.

***Definitions***. The following definitions will be used to classify sepsis cases in the study:

• *Culture-proven bacterial sepsis*: Infants with a positive blood or cerebrospinal fluid culture for a known bacterial pathogen, who present with at least one of the following clinical signs: ill-looking (based on physician assessment), not feeding well (according to parent/caregiver), severe recessions with breathing, convulsions, abdominal distension or lethargy. Infants who present the above-listed criteria and are severely ill (based on physician assessment) are classified as having *s*evere sepsis.

• *Clinical sepsis*: Infants who meet the above-listed criteria in absence of a positive blood culture.

• *Contaminants/unknown*: Growth in the blood culture of multiple bacteria or of strains not commonly considered pathogens (e.g. coagulase-negative Staphylococcus, Micrococcus or Bacillus species) or infants with inconclusive features or microbiology that does not correspond to the clinical picture (e.g. infant remaining clinical well despite microbial culture showing a bacteria that is not covered by the actual antibiotic treatment) ^26, 27^.

*• Non-sepsis controls*: Infants who evolve clinically well without having received antibiotics.

Differences in baseline demographics (gestational age, birth weight, age at presentation, etc.) between the aforementioned groups (sepsis, severe sepsis, clinical sepsis, contaminants, and non-sepsis) will be compared. Significant independent association between culture-proven bacterial and/or clinical sepsis (versus controls), or mortality will be determined using multivariable models adjusting for gestational age or birth weight, sex and age at presentation, plus other significant co-variables. If necessary, we will separately analyze infants who have received antibiotics <4h prior to enrollment from those who have not.

***Matching*.** To identify a gene signature of bacterial sepsis, RNA-Seq will first be run on a subset of infants with culture-proven bacterial sepsis and matched controls. Matching will be performed using a semi-parametric propensity score algorithm ^29^, by identifying the main confounders to the outcome of sepsis. Propensity scores will be estimated from a generalized linear model (GLM) and a nearest neighbor propensity matching with replacement algorithm will be performed to generate a 1:1 match between controls and sepsis cases. Sensitivity of matches will be assessed by using a variable number of potential confounders from the following: sex, gestational age, age, and birth weight.

For RNA-seq, total RNA will be extracted from whole blood using the RiboPure RNA Purification kit. Quantification and quality assessment of total RNA will be performed on an Agilent 2100 Bioanalyzer. Samples with sufficiently high RNA Integrity Number will be considered for sequencing. Poly-adenylated RNA will be captured using the NEBNext Poly (A) mRNA Magnetic Isolation Module. Strand-specific cDNA libraries will be generated from poly-adenylated RNA using the KAPA Stranded RNA-Seq Library Preparation kit and sequenced on a HiSeq 2500 (Illumina; San Diego, CA). Sequence quality will be assessed using FastQC and MultiQC1.8.1. The FASTQ sequence reads will be aligned to the human genome (Ensembl GRCh38.98) using STAR v2.7 and mapped to Ensembl GRCh38 transcripts. Read-counts will be generated using htseq-count (HTSeq 0.11.2-1). Data processing and subsequent differential gene expression will be performed using the latest versions of R and DESeq2 ^30^. Genes with very low counts (with less than 10 counts in the smallest number of biological replicates within each group) and globin transcripts will be filtered out prior to analysis.

***Classifiers*.** We will derive a set of gene classifiers from the RNA-Seq data obtained from matched culture-proven bacterial sepsis and control cases. These classifiers will be derived, first, from differentially expressed genes identified using the Wald statistics test to identify the top 100 differentially expressed genes between groups. Differentially expressed genes will be compared to published literature ( Table 1) to define a final list of curated markers. Additionally, we will apply machine learning approaches to identify potential biomarkers specific to neonatal sepsis from the blood transcriptome. Performance of models from different machine learning approaches ^31, 32^ will be assessed to compare model accuracy, precision and recall. These classifiers will then be applied to culture-negative clinical sepsis cases. ** Given that sepsis outcomes are strongly linked to infants’ sex ^33^, post-natal age ^16, 30^ and other factors such as breastfeeding we will also explore how gene signatures are influenced by these variables, and how sex-related transcript profiles may alter disease severity.

**Table 1.** Other published datasets that included human infants aged <3 months (whole blood).

GEO / EBI accession #	Citation	Type of data	Platform	# infants	Study population
GSE69686	16	Gene array	Affymetrix GeneChip GGh3 Transcriptome Array	68	Newborns admitted to NICU within 24h of sepsis evaluation (US)
GSE25504	12, 13	Gene array	Affymetrix Human Genome U133 Plus 2.0 Array; Illumina Human HT-12 V3.0 expression beadchip; Affymetrix Human Genome U219 Array	62/30/69	Newborns admitted to NICU and i) evaluated for sepsis either i) confirmed or ii) suspected (cases) or iii) “well” (controls) (Scotland)
EMTAB4785	14	Gene array	Affymetrix GeneChip Human 1.0 ST GeneChip	17/19	VLBW neonates with risk factors * and clinical signs of sepsis, or matched controls ^¶^ (Spain)
GSE53471	35	Gene array	Affymetrix Human Genome U219 Array	113	Cord blood from infants born vaginally >36 weeks of gestation (Finland, Estonia, Russia)
GSE64456	27	Gene array	Illumina Human HT-12 V4.0 expression beadchip	279/19	Term infants <60 days evaluated for i) fever (emergency department; cases) or ii) healthy control infants aged 0-6 months (US)
GSE70461	36	Gene array	Affymetrix Human Genome U133 Plus 2.0 Array	20	Newborns <33 weeks in NICU (Norway)
GSE111404 GSE123070	30	RNA-Seq	Illumina HiSeq 2500	60	Healthy term (>36 weeks; >2.5 kg birth weight) (Gambia and Papua New Guinea)
GSE138712	37	RNA-Seq	Illumina HiSeq 2500	18	Preterm neonates born <30 weeks of gestation admitted to the NICU investigated for sepsis in the first 42 days of life (Australia)
GSE40396	38	Gene array	Illumina Human HT-12 V4.0 expression beadchip	4/0/1	Children 2-36 months with i) fever without a source, ii) bacterial infection or iii) well/afebrile, having outpatient surgery (US)
GSE6269	39	Gene array	Affymetrix Human Genome U133A Array Affymetrix Human Genome U133 Plus 2.0 Array Sentrix Human-6 Expression BeadChip	16/0/1/3	Children/adults with i) E. coli, ii) S. aureus, iii) S. pneumonia or iv) influenza A infection (US)

*risk factors: maternal chorioamnionitis, incomplete group B streptococcus (GBS) perinatal prophylaxis, unknown GBS status, rupture of membranes >18 hours, maternal fever during labor, premature labor incompletely treated with antibiotics, neonates with indwelling devices, neonates with recent surgery (<72 hours); ^¶^matched for gestational age, birth weight, gender, ethnicity, mode of delivery, antenatal steroids, postnatal age and “clinical status” GEO: Gene Expression Omnibus EBI: European Bioinformatics Institute; NICU, neonatal intensive care unit; VLBW: Very low birth weight (1,500 g birth weight).

For mNGS, ** both DNA and RNA will be extracted using Zymo Quick DNA/RNA kits and sequenced on an Illumina iSeq platform. We will target an 8 million-reads depth for DNA and 4 million-reads depth for RNA. The data will be analyzed using IDSeq. To determine if bacterial AMR genes can be linked to maternal vaginal flora, we will sequence the DNA from the vaginal swab to determine if the same bacterial AMR genes are present in maternal flora. In an exploratory analysis, we will assess if we can identify the same strain of bacteria, using approaches similar to StrainSifter ^34^.

***Prediction models*.** We will test the ability of clinical variables, but also a limited set of top-discriminating gene markers to predict in-hospital mortality from bacterial sepsis. Clinical variables will include features extracted from the point-of-care vital signs photoplethysmogram and infants’ videos. Univariate analyses will first be carried out to determine their level of association with the mortality outcome. Continuous variables will be assessed for model fit using the Hosmer-Lemeshow test ^40^. Missing data will be imputed by the method of multivariate imputation by chained equations ^41^. Following univariate analysis candidate models will be generated using a step-wise selection procedure minimizing Akaike's Information Criterion (AIC). This method is considered asymptotically equivalent to cross-validation and bootstrapping ^42, 43^. All models generated in this sequence having AIC values within 10% of the lowest value will be considered as reasonable candidates. The final selection of a model will be judged on model parsimony (the simpler the better), availability of the predictors (with respect to minimal resources and cost), and the attained sensitivity. We will aim for a predictive model with a ROC of >0.75–0.8, favoring sensitivity over specificity whenever required. Analyses will be conducted using SAS 9.4 (Carey, NC, USA) and R 3.1.3 (Vienna, Austria; http://www.R-project.org).

### Future data availability

At the end of the study period, de-identified data will be made publicly accessible, following approvals from the University of British Columbia Children’s and Women’s Research Ethics Board, and the National Health Science Research Council of Malawi. A demonstration version of the data collection (no upload to REDCap) of the data collection Android app will be available from the Pediatric Sepsis Data CoLab website: https://dataverse.scholarsportal.info/dataverse/Pedi_SepsisCoLab. RNA-Seq will be deposited with National Center for Biotechnology Information Gene Expression Omnibus.

## Discussion

This study will provide robust epidemiological data in a high risk area for sepsis. This will help address an important global health problem that affects the lives of millions of infants around the world. In the long term, these data could help improve the triaging, diagnosis, and immediate clinical management of young infants with suspected sepsis in both a local and global context. It could potentially also inform more judicial antibiotic use. As antimicrobial resistance is a major rising global health concern, identifying truly septic patients may reduce unnecessary empirical antibiotic use in infants with non-bacterial infections. This study will also directly benefit infants at KCH, by enabling access to standard of care investigations for suspected sepsis and providing human resource support for all infants admitted to the neonatal unit, given the current staffing limitations.

### Strengths

The prospective nature of this study is a major strength. The data collection is informed by rigorous literature reviews ^44, 45^. Studies of neonatal sepsis in LMIC have been mostly retrospective, often starting with case selection by a positive blood culture. In addition, a variety of different definitions for sepsis have been used, without congruence or consistent biological confirmation ^11^. In our study, the use of robust microbiological methods informed by the complementary use of whole blood RNA-Seq may help address these gaps and allow for a more precise estimation of the incidence of sepsis. RNA-seq has not been reported in full-term infants with sepsis and this study will provide these data in LMIC. As the mNGS component of this study will be carried out locally as part of a UNC Project, in Lilongwe, the study will build capacity for using this technology in Malawi, for research and eventually for diagnosis.

### Limitations

There are some limitations in the study protocol. First, the choice of a single regional hospital for recruitment may not be representative of infants assessed for suspected sepsis, for example, in a rural setting. Second, following an infant’s disposition only until discharge will limit an assessment of long-term mortality and morbidity post-discharge. Third, the lack of resources to be able to do even limited investigations for viral pathogens may limit our ability to diagnose non-bacterial sepsis causes. Fourth, we anticipate a number of challenges for this study: some lead investigators are located in geographically remote time zones, which could make troubleshooting and real-time study monitoring more challenging; access to blood sampling outside normal business hours; ensuring a sufficient supply of study supplies and staff in a resource-limited hospital environment; and inconsistent wi-fi/cell network which could complicate data transfer. These challenges will be specifically considered, discussed and addressed throughout the study duration.

## Conclusion

In conclusion, this study protocol aims to address the gap of epidemiological data on the prevalence of sepsis in infants in a LMIC and to contribute to advancing diagnostic precision using RNA-Seq and mNGS. Specific protocols derived for the purpose of this study are outlined followed by a potential data analysis plan. The discussion considers the impact of the study as well as the strengths and limitations. Ultimately, the data generated from the study provides an opportunity to advance the knowledge of sepsis in infants, particularly in LMICs where it has the most substantial impact.

## Data availability

### Underlying data

No underlying data are associated with this article.

### Extended data

Scholars Portal Dataverse: Improving the early diagnosis of neonatal sepsis in Malawi [supplemental material]. https://doi.org/10.5683/SP2/QXBYDX ^22^.

Popescu *et al.* - Supplemental material.pdf contains the following extended data:

- Blood Culture Procedure
- IV Device Access Policy
- Blood Pressure Procedure
- Data Collection Form

Extended data are available under the terms of the Creative Commons Attribution 4.0 International license (CC-BY 4.0).
