# Supplementary material for: Whole blood genome-wide transcriptome profiling and metagenomics next-generation sequencing in young infants with suspected sepsis in a low-and middle-income country: A study protocol
Source: Gates Open Res. 2020 Nov 26;4:139. Originally published 2020 Sep 24. [Version 2] doi: 10.12688/gatesopenres.13172.2 (PMC7783117; doi:10.12688/gatesopenres.13172.2)
Supplement: Peer review history

# Peer review: Reviewer response for version 2

**DOI:** 10.21956/gatesopenres.14417.r30072

**Published:** 2021-01-04

**Authors:** Julia Johnson

**Affiliations:** 1 Division of Neonatology, Department of Pediatrics, Johns Hopkins University, Baltimore, MD, USA

Thank you to the authors for providing such detailed and informative responses to my prior review. All of my questions have been addressed and I have no further concerns or comments. Thank you again for the opportunity to review this protocol, and I look forward to the results of this study.

Is the study design appropriate for the research question?

Yes

Is the rationale for, and objectives of, the study clearly described?

Yes

Are sufficient details of the methods provided to allow replication by others?

Yes

Are the datasets clearly presented in a useable and accessible format?

Not applicable

Reviewer Expertise:

Neonatology, neonatal sepsis, infection prevention and control, healthcare-associated infections, clinical research; US and LMICs

I confirm that I have read this submission and believe that I have an appropriate level of expertise to confirm that it is of an acceptable scientific standard.

# Peer review: Reviewer response for version 2

**DOI:** 10.21956/gatesopenres.14417.r30071

**Published:** 2020-12-15

**Authors:** Andrew Argent

**Affiliations:** 1 Faculty of Health Sciences, University of Cape Town, Cape Town, South Africa

I am happy with the changes that have been made, and am comfortable to approve the article as it now stands following changes.

Is the study design appropriate for the research question?

Yes

Is the rationale for, and objectives of, the study clearly described?

Yes

Are sufficient details of the methods provided to allow replication by others?

Yes

Are the datasets clearly presented in a useable and accessible format?

Yes

Reviewer Expertise:

NA

I confirm that I have read this submission and believe that I have an appropriate level of expertise to confirm that it is of an acceptable scientific standard.

# Response

**Published:** 2020-12-22

**Authors:** Pascal Lavoie

**Affiliations:** BC Women's Hospital & Health Centre, Canada

Thank you

# Peer review: Reviewer response for version 1

**DOI:** 10.21956/gatesopenres.14371.r29656

**Published:** 2020-10-19

**Authors:** Andrew Argent

**Affiliations:** 1 Faculty of Health Sciences, University of Cape Town, Cape Town, South Africa

**General Comments:**

- This is an important study, that has probably been underway for some time at the point of review. It will provide important epidemiological information on sepsis in infants (<3 months of age) in Lilongwe in Malawi.
- The data collected will also open the door to future studies evaluating the potential tools for diagnosis of bacterial infections in small infants. Importantly it may also be possible to evaluate the possibility of excluding bacterial sepsis on the basis of tests done in sick infants. In the longer term that may have important implications for antibiotic stewardship in poorer countries with limited resources for investigation and monitoring of therapy.
- I have not been able to find references in this document to infections such as syphilis. Will this be considered in the study, and is it likely that there would be different responses to intra-uterine infections such as syphilis and more acute bacterial infections?

 

**Specific Comments:**

- Title: Given that this study will be entirely at a single centre in Malawi, I wonder how appropriate it is to title this “in low- and middle- income countries”. It may not be reasonable to assume that these features will be generalizable to such a wide group of countries across the world.
- Introduction: One of the challenges of severe infection and related illness is the overlap of terms such as sepsis / severe infection / bacterial infection. These terms are used interchangeably in multiple settings, when the implications of the different nuances may be significant.
- I would really appreciate it if the authors could: Make it clear that severe infections in neonates could be the consequences of infection with a variety of pathogens including: viruses (e.g. parechovirus infection, herpes infection); bacterial infections (including usual pathogens, but potentially bacteria that are generally associated with normal commensals on the skin and contamination of cultures).
- Para 1: The opening statement of “Sepsis is defined as life-threatening organ dysfunction caused by a dysregulated host response to infection” is made without a reference. Given that the issue of sepsis definitions in infants and children is under review by a variety of working groups, it may be important to provide the reference (from adult sepsis groups).
- Methods - Participants: The authors will be including infants with no indication for antibiotic therapy as control subjects. The protocol states that these infants will be admitted to the study if they need blood sampling for clinical indications. It would be really useful to understand what possible clinical indications there will be for taking blood, and specifically how consent for the study will be taken from the parents of these infants.
- Study procedures: I would appreciate explanation of how active recruitment for the study can be started in June 2018, when the protocol is up for review now.
- Para 4: It is noted that differential counts will be done using coulter counts, however, is there any capacity to check very high counts using manual techniques? In patients with high red cell precursor counts, will white cell counts be corrected for this (I have noted that patients with evidence of extensive hemolysis will have those specimens rejected)?
- Data collection: Given that patients up to 3 months of age will be admitted to the study, how will gestational age be estimated in older infants (I am not concerned that Ballard scores may not be valid after a few days of age)?
- Definitions:
   
  - It would be interesting to consider the items included and not included in the development of the definitions. Factors such as apnea (may overlap with the lethargy, but not necessarily), abnormalities of temperature (either hyper- or hypothermia) have not been included.
  - It is clear that the group being defined as clinical sepsis could be infected with non-bacterial pathogens. However, the authors have not addressed the group who may have been given antibiotics prior to the collection of blood culture specimens (they would be admitted to the study if antibiotics have been given within 4 hours prior to being consented). How likely are patients to fall into this category, and if there are patients in this category, how will they be defined? Is there a reason for a 4-hour cut-off, and how likely are antibiotics prior to culture to adversely impact on culture positivity rates?
  - Clearly infants with factors such as hypoglycemia, dehydration with associated electrolyte and acid-base abnormalities, congenital cardiac problems (probably a very small group of infants) could fall into the clinical sepsis group, but potentially do not have sepsis (as defined by bacterial infection).
- Classifiers: How will “breast-feeding” be classified? What I am addressing is whether the authors will separate total breast-feeding (not other food intake), partial breast-feeding (additional nutrients provided – including oils, porridge etc.) and non-breastfeeding? Will breast-feeding simply be attributed on the basis of the mother’s history?
- Data Analysis:
  - It would be useful to have more information as to how patients will be classified. As an example, if the RNA patterns are compatible with bacterial infection but the cultures are negative (and vice versa) – how will those patients be categorized?
  - It is not clear how other non-bacterial pathogens (including malaria and viral pathogens) will be factored into the analysis and categorization of these patients.
  - To what extent would it be possible during the analysis of all the material collected to consider whether there may be genetic factors in this population that are different to populations in the USA and Europe? To what extent is it possible to evaluate the effect of maternal exposure to pathogens prior to delivery on the infections suffered by the infants?

- Conclusions: This study has substantial strengths, although a potential challenge will be the practicalities of completing the study given the constraints of the particular clinical environment.
- Supplementary Material: It is interesting that bacterial infections of the urinary tract are referred to in the data collection forms, but this is not addressed in the text. Diagnosis of bacterial urinary infections requires close attention to adequacy of specimen collection, and interpretation. It may be important to bring some commentary on this into the text of the main article.

Is the study design appropriate for the research question?

Yes

Is the rationale for, and objectives of, the study clearly described?

Yes

Are sufficient details of the methods provided to allow replication by others?

Yes

Are the datasets clearly presented in a useable and accessible format?

Yes

Reviewer Expertise:

Pediatric critical care; sepsis (particularly in children); low and middle income areas.

I confirm that I have read this submission and believe that I have an appropriate level of expertise to confirm that it is of an acceptable scientific standard, however I have significant reservations, as outlined above.

# Response

**Published:** 2020-11-09

**Authors:** Pascal Lavoie

**Affiliations:** BC Women's Hospital & Health Centre, Canada

We thank the reviewer for their insightful comments which greatly help us improve and clarify important aspects of the protocol. Below are point-by-point responses to each comment:

**Reviewer #2**

 General Comments:

 This is an important study, that has probably been underway for some time at the point of review. It will provide important epidemiological information on sepsis in infants (<3 months of age) in Lilongwe in Malawi. The data collected will also open the door to future studies evaluating the potential tools for diagnosis of bacterial infections in small infants. Importantly it may also be possible to evaluate the possibility of excluding bacterial sepsis on the basis of tests done in sick infants. In the longer term that may have important implications for antibiotic stewardship in poorer countries with limited resources for investigation and monitoring of therapy. I have not been able to find references in this document to infections such as syphilis. Will this be considered in the study, and is it likely that there would be different responses to intra-uterine infections such as syphilis and more acute bacterial infections?

**Reply: **Thank you for this comment. We will consider the exposure and possibility of congenital syphilis in analyses of the transcriptomic data. Routine testing for syphilis and HIV is done in Malawi during antenatal visits. According to the WHO Global AIDS Monitoring in 2018, 1% of women tested on antenatal visits were positive for syphilis. Therefore, we expect few subjects in our cohort to have been exposed during pregnancy.

 Specific Comments:

 Title: Given that this study will be entirely at a single centre in Malawi, I wonder how appropriate it is to title this “in low- and middle- income countries”. It may not be reasonable to assume that these features will be generalizable to such a wide group of countries across the world.

**Reply: **Good suggestion. We have changed the title to: “ **Whole blood genome-wide transcriptome profiling and metagenomics next-generation** **sequencing** **in young infants with suspected sepsis in a low- and middle-income countr y: A study protocol”**. The study is meant to provide the first transcriptome dataset in a LMIC, realizing of course that generalizability will need to be confirmed in future studies in other LMICs.

 Introduction: One of the challenges of severe infection and related illness is the overlap of terms such as sepsis / severe infection / bacterial infection. These terms are used interchangeably in multiple settings, when the implications of the different nuances may be significant.

**Reply:** In neonates and young infants, these are often used interchangeably due to a lack of precise operational definitions. In this article, we have chosen to use the term “sepsis” which is commonly used in the neonatal literature (for lack of better option), but at the same time we understand that this term is somewhat imprecise.

 I would really appreciate it if the authors could: Make it clear that severe infections in neonates could be the consequences of infection with a variety of pathogens including: viruses (e.g. parechovirus infection, herpes infection); bacterial infections (including usual pathogens, but potentially bacteria that are generally associated with normal commensals on the skin and contamination of cultures).

**Reply: **Thank you for the suggestion. We have added this clarification to the introduction.

 Para 1: The opening statement of “Sepsis is defined as life-threatening organ dysfunction caused by a dysregulated host response to infection” is made without a reference. Given that the issue of sepsis definitions in infants and children is under review by a variety of working groups, it may be important to provide the reference (from adult sepsis groups).

**Reply:** We have added a reference from the adult literature as suggested.

 Methods - Participants: The authors will be including infants with no indication for antibiotic therapy as control subjects. The protocol states that these infants will be admitted to the study if they need blood sampling for clinical indications. It would be really useful to understand what possible clinical indications there will be for taking blood, and specifically how consent for the study will be taken from the parents of these infants.

**Reply:** See answer to similar comment from 1 ^st^ reviewer. The need for clinical indication simply refers to the justification for adding an extra blood sample. Informed consent is also obtained from the parent/caregivers of these infants.

 Study procedures: I would appreciate explanation of how active recruitment for the study can be started in June 2018, when the protocol is up for review now.

**Reply:** Thank you for the comment. Indeed, recruitment for our study effectively started in June 2018. We considered publishing the protocol earlier but due to unforeseen delays, we have been unable to do this. In the end, we strongly believe in open access research and in having pre-specified analysis principles and bases before we undertake any analysis of the data, so this is why we insist on publishing this protocol now. This is also in line with our study sponsor’s requirement to make all information about the study available publicly as early as possible, including details in the protocol which would likely not be published elsewhere in the future. Realizing also the importance of structured and comparable datasets for cohort studies, especially in LMICs, we feel very proud to finally see this protocol submitted for review.

 Para 4: It is noted that differential counts will be done using coulter counts, however, is there any capacity to check very high counts using manual techniques? In patients with high red cell precursor counts, will white cell counts be corrected for this (I have noted that patients with evidence of extensive hemolysis will have those specimens rejected)?

**Reply: **Indeed, the UNC lab has the capacity for manual differential on complete blood counts as needed and will be able to provide this data.

 Data collection: Given that patients up to 3 months of age will be admitted to the study, how will gestational age be estimated in older infants (I am not concerned that Ballard scores may not be valid after a few days of age)?

**Reply:** We agree that the Ballard score will not be feasible outside the newborn period. This has been clarified (Page 6). In those circumstances, we rely on available medical records or parent/caregiver recall of information.

 Definitions:

 It would be interesting to consider the items included and not included in the development of the definitions. Factors such as apnea (may overlap with the lethargy, but not necessarily), abnormalities of temperature (either hyper- or hypothermia) have not been included.

**Reply: **We have based our definitions on the WHO list of danger signs, that are also part of the COIN manual in Malawi. Temperature and respiratory rates are recorded automatically on admission. It was very hard for us to conceive that we could accurately capture apneas due to a lack of continuous monitoring or even visual observations due to a profound lack of staff in this setting.

 It is clear that the group being defined as clinical sepsis could be infected with non-bacterial pathogens. However, the authors have not addressed the group who may have been given antibiotics prior to the collection of blood culture specimens (they would be admitted to the study if antibiotics have been given within 4 hours prior to being consented). How likely are patients to fall into this category, and if there are patients in this category, how will they be defined? Is there a reason for a 4-hour cut-off, and how likely are antibiotics prior to culture to adversely impact on culture positivity rates?

**Reply:** The 4-hour cut-off was extensively discussed within our study group. First, based on experience we expected that only a small proportion of infants will have received antibiotic treatment for less than 4 hours prior to initial presentation in this setting. We also considered data suggesting that blood cultures positivity rapidly declines after antibiotic administration (Rand KH et al, Open Forum Infect Dis 2019). Finally, we discussed with our Malawian colleagues that it could be ethically challenging not to offer the study to infants who have received antibiotics for less than 4 hours considering that these infants may benefit from the information provided by blood cultures that were only available as part of this study protocol. Beyond 4 hours, we estimated that the benefit of blood cultures would be sufficiently low to ethically and scientifically justify excluding those infants. This clarification was added in the *Participants* section (Page 4). We have added that: “If necessary, we will separately analyze infants who have received antibiotics prior to enrollment from those who have not” (Definition section; Page 7).

 Clearly infants with factors such as hypoglycemia, dehydration with associated electrolyte and acid-base abnormalities, congenital cardiac problems (probably a very small group of infants) could fall into the clinical sepsis group, but potentially do not have sepsis (as defined by bacterial infection).

**Reply:** Correct.

 Classifiers: How will “breast-feeding” be classified? What I am addressing is whether the authors will separate total breast-feeding (not other food intake), partial breast-feeding (additional nutrients provided – including oils, porridge etc.) and non-breastfeeding? Will breast-feeding simply be attributed on the basis of the mother’s history?

**Reply: **Breastfeeding is classified as exclusive, mixed (with formula) or formula alone and will be recorded form the caregiver’s history. We did not consider other forms of nutrition, as our study population will include mostly young infants in a hospital setting. Virtually 100% infants at KCH are dependent on breastfeeding as there is little formula milk available (as widely advocated by the WHO).

 Data Analysis:

 It would be useful to have more information as to how patients will be classified. As an example, if the RNA patterns are compatible with bacterial infection but the cultures are negative (and vice versa) – how will those patients be categorized?

**Reply: **This is a very good question that we hope will be informed by this study. On way to address this situation is by providing “upper” and “lower” estimates of “bacterial sepsis” and precision assuming the RNA is wrong versus the blood culture is wrong (i.e. false negatives). In absence of satisfying diagnostic gold standard test for bacterial sepsis we can only assess the differences/agreement between these two approaches.

 It is not clear how other non-bacterial pathogens (including malaria and viral pathogens) will be factored into the analysis and categorization of these patients.

**Reply:** We will not perform viral testing in our cohort due to testing availabilities. Malaria testing will be performed if deemed indicated by the treating medical team (e.g. during high seasons), but as per our understanding that although the neonatal mortality from malaria remains high the population incidence is relatively low in this age group (Deribew A et al. Malar J. 2017).

 To what extent would it be possible during the analysis of all the material collected to consider whether there may be genetic factors in this population that are different to populations in the USA and Europe? To what extent is it possible to evaluate the effect of maternal exposure to pathogens prior to delivery on the infections suffered by the infants?

**Reply:** Thank you for the comment. Available transcriptomic data sets from infants suspected of sepsis originate mostly from high-income countries. Therefore, our cohort will be unique for exploring the molecular host responses to infections in a low-income country, and consequently provide the opportunity of comparing the two contexts. Although the mNGS portion of the study may provide focused bacteriological data from the mothers, extensively evaluating the maternal exposure to pathogens will be difficult within the scope of this study.

 Conclusions: This study has substantial strengths, although a potential challenge will be the practicalities of completing the study given the constraints of the particular clinical environment. Supplementary Material: It is interesting that bacterial infections of the urinary tract are referred to in the data collection forms, but this is not addressed in the text. Diagnosis of bacterial urinary infections requires close attention to adequacy of specimen collection, and interpretation. It may be important to bring some commentary on this into the text of the main article.

**Reply:** See similar comment made by the 1 ^st^ reviewer, we added urine cultures to the protocol as suggested.

# Peer review: Reviewer response for version 1

**DOI:** 10.21956/gatesopenres.14371.r29653

**Published:** 2020-10-12

**Authors:** Julia Johnson

**Affiliations:** 1 Division of Neonatology, Department of Pediatrics, Johns Hopkins University, Baltimore, MD, USA

This is an overall well-articulated and clear study protocol for a research study seeking to assess whole blood genome-wide transcriptome profiling and metagenomics NGS in infants with suspected sepsis. The authors lay out the rationale for the study and have selected the appropriate study population and design to answer this question. Thank you for the opportunity to review the protocol of this interesting study.

A few comments:

- Control population could be described in more detail. It seems that only infants with suspected sepsis will be considered for this study, and that the subset of infants who initially present with suspected sepsis but are interpreted as unlikely to have it and not given antibiotics will serve as controls. This does potentially lead to misclassification of infants, including those who initially are fairly well appearing but become ill during admission and are ultimately treated with antibiotics and/or are found to have culture-confirmed bacterial sepsis. How would these infants be handled? An alternative approach would be to include infants who are presenting with other chief complaints, without concern for SBI or sepsis, to serve as true controls.
- Would suggest inclusion of urine samples in young infants, as urinary tract infections are a common cause of serious bacterial infection and associated sepsis in this age group. Urine samples should be obtained by catheter specimen to be of utility.
- The protocol states that rectal swabs will only be obtained in infants weighing greater than 2.5 kg for safety reasons, but do not state what specifically the safety concerns are. Rectal or peri-rectal swabs have been obtained in smaller infants as part of research studies and clinical care, and with appropriate procedures, this should not be an issue. Avoidance in very small extremely preterm infants may be indicated, but this does not seem to be the likely population for this study. If there is concern, perhaps stool samples could be obtained instead in infants below threshold of a weight or age cutoff, if available. 
- It may be beneficial to describe what is known about neonate/infant sepsis at the study site based on microbiology data, which would help the reader interpret whether the described microbiologic procedures (and standard antibiotic therapy) are likely to capture the most common causes of sepsis in this population. This may not be necessary for the protocol but should be included in a future manuscript.
- This may exceed the scope of the study, but to adequately capture etiologic pathogens of infants with clinical sepsis without identified bacterial pathogen, the authors could consider adding at least a limited investigation for viral pathogens.
- Would recommend review of this protocol by an additional reviewer with specific expertise in NGS.

Comments on supplemental materials:

- Would recommend providing specific time recommended for betadine/iodine to air dry (as provided for alcohol), as inadequate time to dry is a common lapse in IPC practices for blood culture collection that leads to contamination.
- Would specify acceptable sites for blood draws.
- For BP measurements, it currently states to recheck if low in 30 minutes. Depending on how low the BP is, this may be a critical finding and waiting 30 minutes could be life-threatening. Recommend rechecking within 5 minutes or less to assess validity of finding. Also, consider providing BP norms by age/weight to assist providers, unless these are readily known.
- For the birth history, it states to enter full term as 40 weeks. Full term includes 37-40 weeks. This granularity of data may not be needed, but if descriptive statistics, such as median GA, are performed, inclusion of 40 weeks for all term infants may be misleading. Consider creating a separate question of preterm (yes/no) and using a GA question as follow-up for only preterm instead.
- For type of delivery, what does "bre" stand for? Breech? Would this mean breech extraction/vaginal delivery or C-section for breech? Would expand most abbreviations to avoid any confusion.
- Describe what resuscitation at birth means - any intervention at all by medical team? Only need for respiratory intervention such as oxygen, PPV, or intubation, or need for compressions? Unclear, for example, whether need for suctioning would be considered needing resuscitation for this form's purpose.
- Number of days in hospital: consider collection of precise admission date and discharge date to avoid any errors in calculation or inadvertent incorrect interpretation of partial days.

Is the study design appropriate for the research question?

Yes

Is the rationale for, and objectives of, the study clearly described?

Yes

Are sufficient details of the methods provided to allow replication by others?

Yes

Are the datasets clearly presented in a useable and accessible format?

Not applicable

Reviewer Expertise:

Neonatology, neonatal sepsis, infection prevention and control, healthcare-associated infections, clinical research; US and LMICs

I confirm that I have read this submission and believe that I have an appropriate level of expertise to confirm that it is of an acceptable scientific standard, however I have significant reservations, as outlined above.

# Response

**Published:** 2020-11-09

**Authors:** Pascal Lavoie

**Affiliations:** BC Women's Hospital & Health Centre, Canada

We thank the reviewer for her insightful comments which greatly help us improve and clarify important aspects of the protocol. Below are point-by-point responses to each comment:

**Reviewer #1**

 This is an overall well-articulated and clear study protocol for a research study seeking to assess whole blood genome-wide transcriptome profiling and metagenomics NGS in infants with suspected sepsis. The authors lay out the rationale for the study and have selected the appropriate study population and design to answer this question. Thank you for the opportunity to review the protocol of this interesting study. A few comments:

 Control population could be described in more detail. It seems that only infants with suspected sepsis will be considered for this study, and that the subset of infants who initially present with suspected sepsis but are interpreted as unlikely to have it and not given antibiotics will serve as controls. This does potentially lead to misclassification of infants, including those who initially are fairly well appearing but become ill during admission and are ultimately treated with antibiotics and/or are found to have culture-confirmed bacterial sepsis. How would these infants be handled? An alternative approach would be to include infants who are presenting with other chief complaints, without concern for SBI or sepsis, to serve as true controls.

**Reply:** Our initial plan was to recruit infants with other chief complaints without concern for SBI. However, the Malawi IRB felt strongly that this control group was not scientifically adequate and requested that we include in our control group “only infants in whom sepsis was sufficiently unlikely so that antibiotics were not to be started”. In the end, we are collecting antibiotic administration during the hospital stay so we will have the possibility of excluding infants who initially are fairly well appearing but become ill during admission from the control group. Therefore, the risk of misclassification is likely to be extremely low.

 Would suggest inclusion of urine samples in young infants, as urinary tract infections are a common cause of serious bacterial infection and associated sepsis in this age group. Urine samples should be obtained by catheter specimen to be of utility.

**Reply:** As suggested by the reviewer we have included it, but it is likely that this will not be available in all infants. Urine cultures were part of our initial protocol, but the feasibility in this setting was uncertain.

 The protocol states that rectal swabs will only be obtained in infants weighing greater than 2.5 kg for safety reasons, but do not state what specifically the safety concerns are. Rectal or peri-rectal swabs have been obtained in smaller infants as part of research studies and clinical care, and with appropriate procedures, this should not be an issue. Avoidance in very small extremely preterm infants may be indicated, but this does not seem to be the likely population for this study. If there is concern, perhaps stool samples could be obtained instead in infants below threshold of a weight or age cut-off, if available.

**Reply:** We have reworded this section to make this clearer (Page 5). The safety concern is about taking an extra research blood sample in smaller, potentially ill babies, i.e. not the swab itself. This was expressed by our clinician colleagues at the Kamuzu Central Hospital. Culturally, they felt that this may be difficult to accept for parents. In making the decision to exclude babies <2.5 kg, we considered that the main objective of the mNGS component of the study is to provide feasibility data so in this context it was not clearly warranted to include smaller babies. The rectal and vaginal swabs are to be done and analyzed in parallel to the blood sample and therefore were not planned to be done in babies <2.5kg for the same reasons.

 It may be beneficial to describe what is known about neonate/infant sepsis at the study site based on microbiology data, which would help the reader interpret whether the described microbiologic procedures (and standard antibiotic therapy) are likely to capture the most common causes of sepsis in this population. This may not be necessary for the protocol but should be included in a future manuscript.

**Reply: **Thank you for the comment. We have added this information to the Study size method section (Page 6-7). Blood cultures are not routinely done for neonates suspected of sepsis at KCH, so it is challenging to have baseline microbiological data. Our study will provide blood cultures for the infants suspected of sepsis enrolled in the study and therefore detail the microbiological landscape in this population. However, based on studies done at the Queen Elizabeth Central Hospital, Blantyre, Malawi (also a regional referral centre), we expect a relatively high incidence or Gram-negative pathogens displaying resistance patterns to first-line antimicrobials (Iroh Tam et al, Clin Infect Dis 2019). We will also explore resistance to antibiotics for pathogens detected in our cohort.

 This may exceed the scope of the study, but to adequately capture etiologic pathogens of infants with clinical sepsis without identified bacterial pathogen, the authors could consider adding at least a limited investigation for viral pathogens.

**Reply:** Unfortunately, the logistics of running viral samples in this setting is complex, precluding this as a possibility. There are simply no adequate resources to do this. We have added this to the limitation section of the discussion.

 Comments on supplemental materials:

 Would recommend providing specific time recommended for betadine/iodine to air dry (as provided for alcohol), as inadequate time to dry is a common lapse in IPC practices for blood culture collection that leads to contamination.

**Reply: **This has been reworded in the Supplemental Material, reuploaded on the dataverse. In fact, during our on-site study training, we intended to applying the same scrubbing and air drying times for betadine/iodine as for alcohol.

 Would specify acceptable sites for blood draws.

**Reply:** For practical and safety reasons, we entrust the decision to the clinical team at the KCH, as they are best placed to evaluate the infant’s clinical status, medical management and level of comfort with the procedure of the healthcare worker preforming the blood draw.

 For BP measurements, it currently states to recheck if low in 30 minutes. Depending on how low the BP is, this may be a critical finding and waiting 30 minutes could be life-threatening. Recommend rechecking within 5 minutes or less to assess validity of finding. Also, consider providing BP norms by age/weight to assist providers, unless these are readily known.

**Reply:** The reviewer is absolutely right commenting that blood pressure q30 min may be too long to provide safe clinical care in a sick baby. However, this is an observational study that aims to document, not intervene. At KCH, measuring blood pressure in neonates has not been integrated in routine clinical care, in absence of proper equipment. Our study provides the first blood pressure monitors for newborns, as well as training for the medical team on their use. We also provide support for the clinical team with BP targets if needed. However, the goal here is to begin integrating vital signs monitoring for infants, considering the challenges in managing low BP in LMICs (lack of continuous monitoring or inotropic support etc.). In deciding to suggest repeating blood pressure 30 minutes after, we also considered that nurses are profoundly lacking and overstretched in this setting, and IV infusions are largely unavailable.

 For the birth history, it states to enter full term as 40 weeks. Full term includes 37-40 weeks. This granularity of data may not be needed, but if descriptive statistics, such as median GA, are performed, inclusion of 40 weeks for all term infants may be misleading. Consider creating a separate question of preterm (yes/no) and using a GA question as follow-up for only preterm instead.

**Reply: **Age is recorded as number of completed gestational weeks (the electronic form actually encourages the data collector to enter the exact GA, which we realize is not obvious looking at the material provided).

 For type of delivery, what does "bre" stand for? Breech? Would this mean breech extraction/vaginal delivery or C-section for breech? Would expand most abbreviations to avoid any confusion.

**Reply: **That is correct, it stands for breech vaginal delivery. We have had to abbreviate the term in the case report form due to space constraints in the data collection forms.

 Describe what resuscitation at birth means - any intervention at all by medical team? Only need for respiratory intervention such as oxygen, PPV, or intubation, or need for compressions? Unclear, for example, whether need for suctioning would be considered needing resuscitation for this form's purpose.

**Reply: **Correct, resuscitation is defined as any intervention by medical team. Due to a lack of formal chart documentation this information will often be collected from parents/caregivers who have no idea what these different interventions are. Resuscitation include suctioning, bag and mask ventilation and chest compressions. Presently, neonates are not intubated at KCH. Suctioning alone is not considered resuscitation.

 Number of days in hospital: consider collection of precise admission date and discharge date to avoid any errors in calculation or inadvertent incorrect interpretation of partial days.

**Reply:** As per our IRB, it was not possible to collect “dates” (for privacy reasons). We collect the admission date in the infant’s chart, and the duration of hospitalization is calculated at the moment of discharge. We trust that this method will be able to provide accurate hospital stays.
